# Continuous Flow Alcoholysis of Dialkyl *H*-Phosphonates with Aliphatic Alcohols

**DOI:** 10.3390/molecules23071618

**Published:** 2018-07-03

**Authors:** Erika Bálint, Ádám Tajti, Nóra Tóth, György Keglevich

**Affiliations:** Department of Organic Chemistry and Technology, Budapest University of Technology and Economics, 1521 Budapest, Hungary; tajti.adam@mail.bme.hu (Á.T.); toth.nora@mail.bme.hu (N.T.)

**Keywords:** dialkyl *H*-phosphonates, alcoholysis, transesterification, microwave, continuous flow reactor

## Abstract

The continuous flow alcoholysis of dialkyl *H*-phosphonates by aliphatic alcohols in the absence of a catalyst was elaborated using a microwave (MW) reactor equipped with a flow cell. By the precise control of the reaction conditions, the synthesis could be fine-tuned towards dialkyl *H*-phosphonates with two different and with two identical alkyl groups. In contrast to the “traditional” batch alcoholysis, flow approaches required shorter reaction times, and the products became available at a larger scale.

## 1. Introduction

Flow chemistry induced an inevitable revolution in the field of chemical transformations [1]. In modern chemistry, reactions have to be strictly controlled, which requires more and more precise synthetic techniques. In contrast to the “traditional” batch reactions, flow approaches create a significantly different processing environment, which enables a more efficient control of the reaction conditions (such as the reaction time or temperature). Due to this, flow protocols may be significantly better with respect to purity, selectivity and yields, as compared to batch approaches [2].

In microwave (MW) chemistry, the continuous flow technique represents a special importance. Although applications of the MW technique proved to be useful in many chemical transformations, the scale-up of MW-assisted reactions means a challenge due to the limited geometry of the MW devices [3,4,5,6]. One possibility to solve this problem is the use of continuous flow MW reactors, where the reaction mixture flows through an irradiated flow cell. The MW unit can be of a similar size used in batch mode. During the last decade, the advantages of the applications of continuous flow MW reactors were reported in certain transformations; however, in most cases, the usefulness of the occasional devices may be questionable, as non-professional MW reactors do not allow reproductions, and what is more important, the temperatures were not reported [7,8].

Esters represent a fundamental family among organic compounds. Esters of carboxylic acids may be important intermediates, solvents or products in organic chemistry [9].

The most common preparations of simple carboxylic esters comprise the acid-catalyzed reaction of a carboxylic acid with alcohol (Fischer esterification) and the reaction of an ester with alcohol (alcoholysis) [10]. Continuous flow Fischer esterifications may be carried out in systems containing a packed catalyst bed [11,12,13,14,15,16,17,18,19,20] or a heated coil [21,22], as well as in microreactors [23]. In a few cases, the esterifications were performed in continuous flow MW systems based on professional MW reactors [24,25,26] or in household MW ovens [27,28]. The continuous flow alcoholysis of carboxylic esters may also be performed in reactors equipped with a catalyst bed [29] or a heated coil [21,30]. The alcoholysis is also of great importance in biodiesel production, the area of which was recently summarized by Lee and co-workers [31].

Organophosphorus esters are also of great importance [32]. Dialkyl esters of phosphorous acid (dialkyl *H*-phosphonates) are widely applied building blocks in syntheses [33]. They are important starting materials of the Kabachnik–Fields condensations and the aza-Pudovik reactions [34] (towards α-aminophosphonates), the Pudovik reactions [35] (resulting in the formation of α-hydroxyphosphonates) and further organophosphorus transformations, such as the Hirao reaction [36] and the phospha-Michael addition [37]. Dialkyl *H*-phosphonates bearing different alkoxy groups on the phosphorus atom are valuable intermediates for P-chiral organophosphorus derivatives [38,39].

**Scheme 1:**
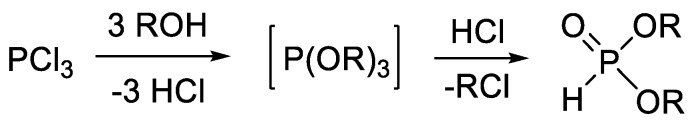
Synthesis of dialkyl *H*-phosphonates from phosphorus trichloride.

The industrial synthesis of dialkyl *H*-phosphonates is comprised of the reaction of phosphorus trichloride with alcohols (Scheme 1) [40,41,42,43,44,45]. Although this transformation is convenient, efficient and may be scaled up, it requires a solvent, and the liberating HCl decreases the atom efficiency. It is also a disadvantage that the synthesis of derivatives with different alkyl groups is not possible.

**Scheme 2:**
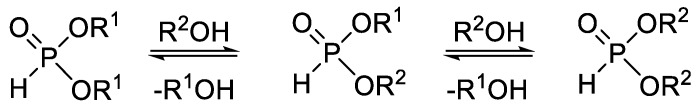
Synthesis of dialkyl *H*-phosphonates by alcoholysis.

Another possibility for the preparation of dialkyl *H*-phosphonates is alcoholysis (Scheme 2), which provides the products in good yields [46,47,48,49,50,51,52,53,54]. The only by-product is the leaving alcohol. The relatively high temperature and the need for the high excess of the alcohol mean disadvantages.

**Scheme 3:**
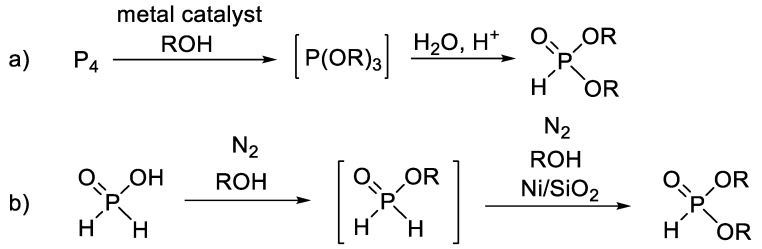
Miscellaneous synthesis of dialkyl *H*-phosphonates.

Besides the two main routes mentioned above, there are a few less important methods for the synthesis of dialkyl *H*-phosphonates. Such protocols are the oxidative reaction of elemental phosphorus with alcohols (Scheme 3a) [55,56,57,58,59,60] and the Ni-catalyzed oxidation of hypophosphorous esters (Scheme 3b) [61].

The synthesis of dialkyl-*H*-phosphonates bearing different alkyl groups is not easy and requires special methods (Table 1).

Phosphites with different alkyl groups may be synthesized by the partial alcoholysis of dialkyl *H*-phosphonates (Table 1, Entry 1). The reaction conditions must be controlled strictly to avoid complete transesterification.

Dialkyl *H*-phosphonates with two different alkyl groups may be obtained by the controlled O–C cleavage of dialkyl *H*-phosphonates (Table 2, Entries 2 and 3). In the first step of the first method, a phosphite-ammonium salt is prepared, which is then alkylated with alkyl iodides (Table 2, Entry 2), or with alcohols in the presence of pivaloyl chloride and pyridine (Table 2 entry 3) to furnish the target compounds selectively. A serious drawback of these transformations is the low atom efficiency.

Dialkyl *H*-phosphonates bearing different alkoxy groups can also be obtained in the reaction of alkyl dichlorophosphites and alcohols. The primarily formed trialkyl phosphite is cleaved by a part of the hydrochloric acid formed (Table 2, Entry 4).

From these synthetic methods, the alcoholysis may be flow compatible. This solvolytic reaction may be carried out without any catalyst by fine-tuning the reaction conditions, to afford mixed or fully-transesterified dialkyl phosphites as the predominant component.

The alcoholysis of dialkyl *H*-phosphonates in a batch MW reactor was investigated by us previously (Table 2) [52]. The reaction of dialkyl *H*-phosphonates (**A**) with aliphatic alcohols under mild conditions (lower alcohol excess and lower temperature) afforded the derivatives with different alkyl groups (**B**) as the main products, while in the case of applying higher excess of alcohol and higher temperature, the fully-transesterified dialkyl *H*-phosphonates (**C**) predominated in the mixture.

In this paper, we aimed at elaborating an MW-assisted continuous flow method for the alcoholysis of dialkyl *H*-phosphonates. Our purpose was to find the optimum conditions for the formation of dialkyl *H*-phosphonates bearing different alkyl groups and for the fully-transesterified phosphites in a flow MW reactor. Furthermore, we wished to prepare new dialkyl *H*-phosphonates with two different alkyl groups, which may be valuable building blocks of chiral organophosphorus compounds.

## 2. Results and Discussion

**Figure 1:**
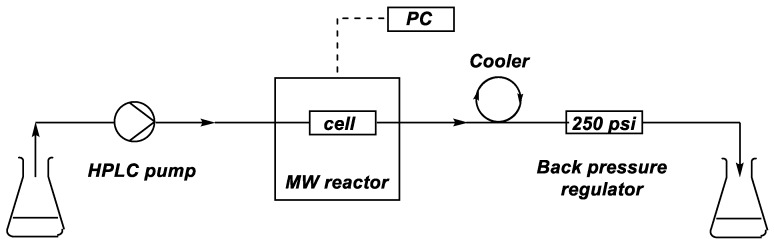
Schematic drawing of the continuous flow system developed.

**Figure 2:**
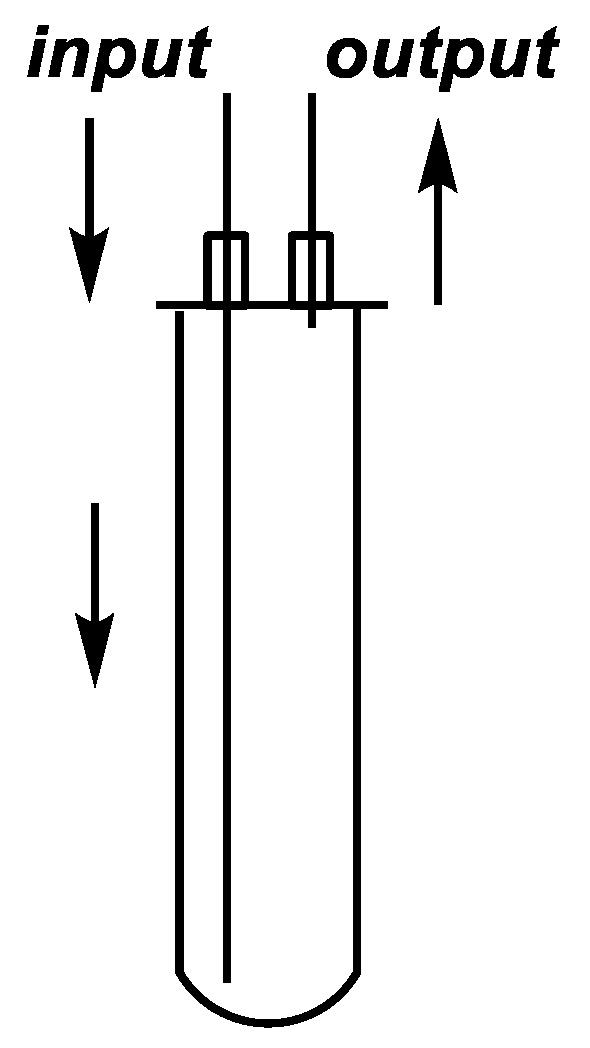
Sketch of the continuous flow cell.

In the first case, the alcoholysis of dimethyl *H*-phosphonate (DMP) (**1**) with *n*-butanol was investigated in the absence of any catalyst, in a CEM^®^ (Matthews, NC, USA) MW reactor equipped with a commercially available CEM^®^ continuous flow cell (Figure 1 and Figure 2). The mixture of DMP (**1**) and a 25-fold excess of *n*-butanol was fed into the reactor by an HPLC pump at a flow rate of 0.1–1.4 mL/min (corresponding to residence times of 60–5 min, respectively). The temperature was monitored and controlled by an IR sensor. The mixture leaving the reactor was cooled down using a spiral-like cooler and was passed through a back pressure regulator operating at 250 psi. Consecutive fractions of the leaving mixture were analyzed by GC measurements in order to determine the composition and to identify the stationary operation.

**Figure 3:**
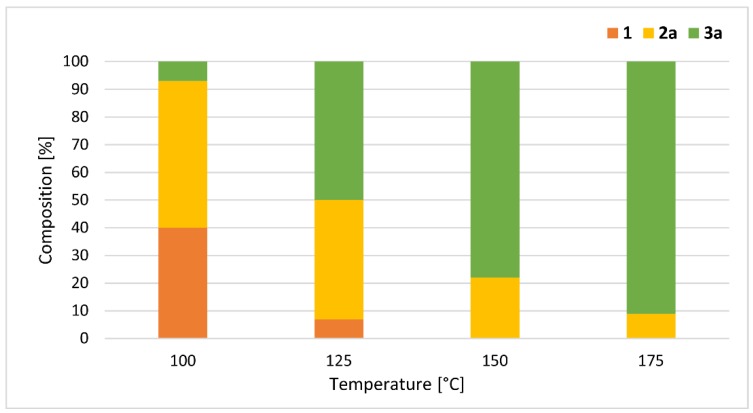
Composition of the reaction mixtures of the MW-assisted alcoholysis of DMP with *^n^*BuOH at different temperatures at a residence time of 30 min (Table 3 entries 3 and 7–9).

The reaction conditions and the results are summarized in Table 3. To find the optimum conditions for the formation of *n*-butyl methyl *H*-phosphonate (nBMP) (**2a**) and *n*-dibutyl *H*-phosphonate (DnBP) (**3a**), the alcoholysis was carried out applying different temperatures and residence times. Performing the reaction at 100 °C, at a flow rate of 1.4 mL/min (with a residence time of 5 min), the conversion was only 26%, and the proportion of nBMP (**2a**) was 25%, along with 1% of DnBP (**3a**) (Table 3, Entry 1). Using a flow rate of 0.45 mL/min (with a residence time of 15 min), the starting DMP (**1**) was still the main component of the leaving mixture; however, the proportion of nBMP (**2a**) was 44% (Table 3, Entry 2). Increasing the residence time to 30 min (by decreasing the flow rate to 0.25 mL/min), the ratio of mixed *H*-phosphonate (**2a**) was 9% higher (53%) (Table 3, Entry 3). Applying a longer residence time of 45 or 60 min (flow rate of 0.15 or 0.10 mL/min), the proportion of phosphite **2a** was somewhat lower (50% or 47%), and the ratio of DnBP (**3a**) increased to 26% or 35%, respectively (Table 3, Entries 4 and 5). The maximum proportion of nBMP (**2a**) (53%) was obtained applying a reaction time of 30 min at 100 °C (Table 3, Entry 3). Increasing or decreasing the residence time at the same temperature resulted in lower ratio of product **2a** (Table 3, Entries 1, 2 and 4, 5, respectively). In a comparative thermal experiment at 100 °C at a residence time of 30 min, the conversion was only 15% (Table 3, Entry 6). Next, the effect of the reaction temperature was investigated at a residence time of 30 min (Table 3, Entries 6–8) (Figure 3). At 125 °C, the mixture was comprised of 7% of DMP (**1**), 43% of nBMP (**2a**) and 50% of DnBP (**3a**) (Table 3, Entry 7). Increasing the temperature to 150 °C, the conversion was complete, and the fully-transesterified product (**3a**) predominated in a proportion of 78% (Table 3, Entry 8). Performing the alcoholysis at 175 °C, the ratio of DnBP (**3a**) was 91% (Table 3, Entry 9). Carrying out the reaction at a longer residence time (45 min) and/or higher excess of the *^n^*BuOH (50 equiv), the composition did not change. Applying common heating at 175 °C, the reaction was not complete, and the ratio of product **3a** was 8% lower, than under MW conditions (Table 3, Entry 10). It can be observed that the difference between MW-assisted and conventionally-heated experiments was significant at 100 °C, while significantly smaller at 175 °C. After column chromatography, nBMP (**2a**) was obtained in a yield of 48%, while DnBP (**3a**) was isolated in a yield of 88% (Table 3, Entries 3 and 9).

Comparative batch experiments were also carried out in the DMP (**1**)—*^n^*BuOH model reaction at 100 °C. The alcohol excess (25 equiv) and the reaction time range studied (5–60 min) were the same as in the flow approaches. The batch results are listed in Table 4. Similarly to the flow experiments, the composition was highly reaction time-dependent.

**Figure 4:**
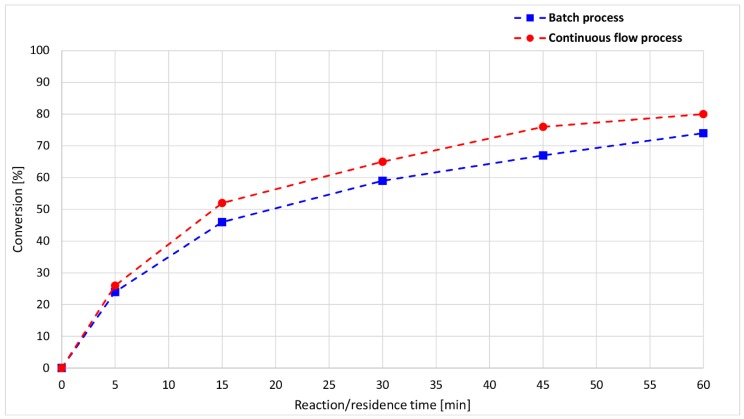
Reaction/residence time-dependence of the conversion of the alcoholysis of DMP with *^n^*BuOH at 100 °C.

Comparing the conversions of the flow and batch processes, the two plots show a similar shape; however, after a reaction time of 15 min, the batch conversions were 5–10% lower than the flow results (Figure 4).

**Figure 5:**
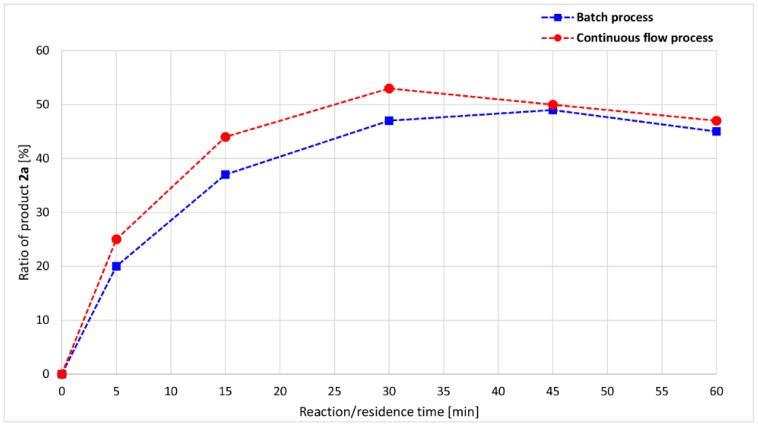
Reaction/residence time-dependence of the product **2a** ratio in the alcoholysis of DMP with *^n^*BuOH at 100 °C.

A more significant difference may be observed if the ratio of product **2a** is examined separately (Figure 5). Maximum points are present on both curves: at 30 min and 53% in the flow experiments and at 45 min 49% in the case of the batch approaches. Based on the comparison, the flow reaction reaches the optimum point of phosphite **2a** under a shorter time, with a higher ratio.

Based on the experiences above, the alcoholysis of DMP (**1**) was also carried out with *n*-propanol, *i*-butanol, as well as *n*-pentanol at 100 °C and 175 °C with a residence time of 30 min. In all cases, a mixture containing DMP (**1**) and 25 equivalents of the corresponding alcohol was passed through the continuous flow system. The data shown in Table 4 are concerned to the stationary operation. In the reaction of DMP (**1**) with *^n^*PrOH at 100 °C, the leaving mixture contained 45% of unreacted DMP (**1**), 46% of *n*-propyl methyl *H*-phosphonate (PrMP) (**2b**) and 9% of *n*-dipropyl *H*-phosphonate (DPrP) (**3b**) (Table 5, Entry 1). At a higher temperature of 175 °C, the fully-transesterified product (**3b**) was the major component (90%) (Table 5, Entry 2). The outcome of the alcoholysis of DMP (**1**) with *^i^*BuOH and *^n^*PentOH was similar (Table 5, Entries 3–6) However, in the case of the experiments carried out at 100 °C, the ratio of *i*-butyl methyl *H*-phosphonate (iBMP) (**2c**) and *n*-pentyl methyl *H*-phosphonate (PeMP) (**2d**) was above 50% (Table 5, Entries 3 and 5). After column chromatography, the corresponding mixed phosphonates (**2b**, **2c** and **2d**) were obtained in yields of 42–51% (Table 5, Entries 1, 3 and 5), and the fully-transesterified products (**3b**, **3c** and **3d**) were isolated in yields of 85–88% (Table 5, Entries 2, 4 and 6).

The next model was the continuous flow alcoholysis of diethyl *H*-phosphonate (DEP) (**4**) (Table 6). Based on the experiences from the transesterification of DMP (**1**), the reactions were carried out using 25 equivalents of the alcohols at a flow rate of 0.25 mL/min (or at a residence time of 30 min). First, the alcoholysis of DEP (**4**) by *^n^*BuOH was investigated. Performing the reaction at 100 °C, the unreacted DEP (**4**) was the main component along with 34% of *n*-butyl ethyl *H*-phosphonate (nBEP) (**5a**) and 8% of DBP (**3a**) (Table 6, Entry 1). At a higher temperature of 125 °C, the ratio of nBEP (**5a**) was 42%, and after column chromatography, compound **5a** could be isolated in a yield of 38% (Table 6, Entry 2). Increasing the temperature to 150 °C and 175 °C, the proportion of **5a** was decreased, while that of DBP (**3a**) increased (Table 6, Entries 3 and 4). In the latter case, the alcoholysis was complete, and the DBP (**3a**) was obtained in a yield of 85% (Table 6, Entry 4). The transesterification of DEP (**4**) by *^n^*PrOH at 125 °C took place similarly as the alcoholysis by *^n^*BuOH, and the *n*-propyl ethyl *H*-phosphonate (PrEP) (**5b**) was obtained in a yield of 36% (Table 6, Entries 2 and 5). At 175 °C, the conversion was not complete, and the resulting mixture contained only 74% of DPrP (**3b**) besides 4% of unreacted DEP (**4**) and 22% of PrEP (**5b**) (Table 6, Entry 6). To obtain a higher proportion of product **3b**, the temperature was increased to 200 °C. Hence, phosphite **3b** was formed in 84% (Table 6, Entry 7). In the case of *^i^*BuOH, the tendency was similar, and the highest amount of mixed phosphonate (**5c**) was formed at 125 °C, while at 175 and 200 °C, the fully-transesterified product (**3c**) predominated (Table 6, Entries 8–10). Using PentOH at 125 °C, the pentyl ethyl *H*-phosphonate (PeEP) (**5d**) was the main component in the departing mixture, from which it was isolated in a yield of 40% (Table 6, Entry 11). For the preparation of dipentyl *H*-phosphonate (DPeP) (**3d**), the application of 175 °C was enough, and compound **3d** was obtained in a yield of 80% (Table 6, Entry 12).

The catalyst-free alcoholysis of dialkyl *H*-phosphonates with various aliphatic alcohols was carried out efficiently in a continuous flow MW reactor. Applying a residence time of 30 min at different temperatures, the alcoholysis could be fine-tuned towards the dialkyl *H*-phosphonates with two different or with two identical alkyl groups. In the case of the reaction studied, the dimethyl phosphite proved to be the more reactive starting material. After almost complete conversions, the (RO)_2_P(O)H species formed by transesterification were obtained in yields above 85%. The ratio of (RO)(R’O)P(O)H derivatives with two different alkyl groups was around 50% in all mixtures, affording the target compounds in a yield range of ca*.* 40–50%. By the method developed, a series of mixed dialkyl *H*-phosphonates, which are valuable building blocks for the preparation of chiral organophosphorus derivatives, was synthesized.

## 3. Materials and Methods

### 3.1. General

GC measurements were performed on an HP5890 Series 2 GC-FID chromatograph, using a 15 m × 0.18 mm Restek, Rtx-5 column with a film layer of 0.20 μm. The temperature of the column was initially held at 40 °C for 1 min, followed by programming at 25 °C/min up to 300 °C and a final period at 300 °C (isothermal) for 10 min. The temperature of the injector was 290 °C and of the FID detector was 300 °C. The carrier gas was N_2_.

GC-MS measurements were performed on an Agilent 6890 N-GC-5973 N-MSD chromatograph, using a 30 m × 0.25 mm Restek, Rtx-5SILMS column with a film layer of 0.25 μm. The initial temperature of the column was 45 °C for 1 min, followed by programming at 10 °C/min. up to 310 °C and a final period at 310 °C (isothermal) for 17 min. The temperature of the injector was 250 °C. The carrier gas was He, and the operation mode was splitless.

High resolution mass spectrometric measurements were performed using a TripleTOF 5600+ mass spectrometer in positive electrospray mode.

The ^13^C- and ^1^H-NMR spectra were obtained in CDCl_3_ solution on a Bruker DRX-500 spectrometer operating at 125.7 and 500.1 MHz, respectively. The ^13^C and ^1^H chemical shifts are referred to TMS. ^31^P-NMR spectra were obtained on a Bruker AV-300 spectrometer at 121.5 MHz. Chemical shifts are downfield relative to 85% H_3_PO_4_.

### 3.2. Equipment

**Figure 6:**
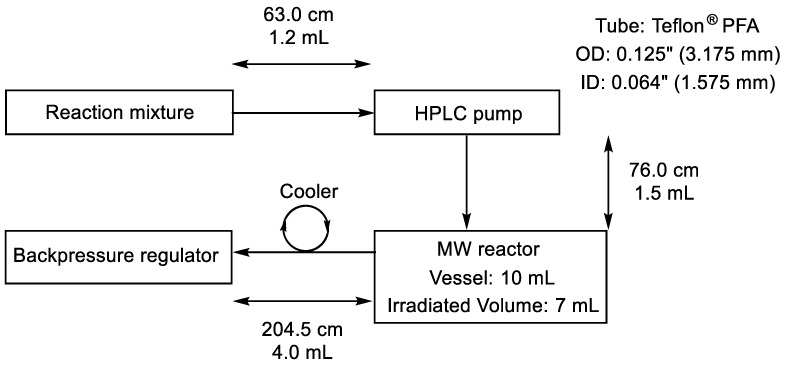
Design parameters of the continuous flow system.

The continuous flow reactions were performed in a self-developed continuous flow system comprising a 300-W CEM^®^ Discover-focused microwave reactor equipped with a CEM^®^ 10-mL Flow Cell Accessory continuous flow unit (irradiated volume 7 mL), a Gilson 305 HPLC pump, an HPLC backpressure regulator with a 250-psi (17.2 bar) cartridge and a cooler. Teflon^®^ (Wilmington, DE, USA) PFA tubes with an outside diameter of 0.125” (3.175 mm) and an inside diameter of 0.064” (1.575 mm) were used. The exact lengths and volumes of each tube part are shown in Figure 6. All of the tubes, screws and ferrules applied were fully compatible with a regular HPLC system.

### 3.3. General Procedure for the Continuous Flow Alcoholysis of Dialkyl H-Phosphonates

A mixture of 25.0 mmol of the dialkyl *H*-phosphonate (2.7 mL of dimethyl *H*-phosphonate, 3.9 mL of diethyl *H*-phosphonate) and 25 equivalents, 0.625 mol of alcohol (47 mL of *n*-propanol, 57 mL of *n*-butanol, 58 mL of *i*-butanol, 68 mL of *n*-pentanol) was homogenized by stirring for 5 minutes at 25 °C. The reactor was flushed with 20 mL of the mixture with a flow rate of 10 mL/min at 25 °C and 17 bar. Next, under the same pressure, the flow rate was set to the desired value, and the vessel was irradiated with a power of 45–300 W for 3–5 min, until the temperature reached the desired value, then the power was controlled automatically by the software of the MW reactor. The flow rate and the necessary power at stationary operation can be seen in Table 3, Table 5 and Table 6. The operation was regarded as the steady state on the basis of the results of the GC measurements. The products were separated by column chromatography using silica gel as the absorbent and ethyl acetate as the eluent. Yields were calculated on the basis of the weights obtained after separation and evaporation taking into consideration the quantity of the dialkyl *H*-phosphonate fed in during a given time. The ^31^P-NMR and mass data for the dialkyl *H*-phosphonates are listed in Table 7.

### 3.4. General Procedure for the Comparative Batch Experiments

A mixture of 0.5 mmol (0.05 mL) of dimethyl *H*-phosphonate and 25 equivalents, 12.5 mmol (1.1 mL) of *n*-butanol was heated at 100 °C in a vial in the MW reactor for 5–60 min as shown in Table 4. The volatile components were removed in vacuum, and the residual oil was analyzed by GC.

*Methyl propyl H-phosphonate* (**2b**): Yield: 42%; ^31^P-NMR and HRMS (see Table 7); ^13^C-NMR (CDCl_3_) δ 10.0 (*C*H_3_CH_2_), 23.8 (*J* = 6.2, CH_2_), 52.0 (*J* = 5.8, *C*H_3_O), 67.1 (*J* = 6.0, *C*H_2_O); ^1^H-NMR (CDCl_3_) δ 0.98 (t, *J_HH_* = 7.4, 3H, C*H*_3_CH_2_), 1.65–1.82 (m, 2H, CH_2_), 3.78, (d, *J_PH_* = 11.9, 3H, CH_3_O) 3.97–4.12 (m, 2H, CH_2_O), 6.79 (d, *J_PH_* = 695.4, 1H, PH).

*i-Butyl methyl H-phosphonate* (**2c**): Yield: 46%; ^31^P-NMR and HRMS (see Table 7); ^13^C-NMR (CDCl_3_) δ 18.6 (*J* = 3.1, *C*H_3_CH), 29.2 (*J* = 6.4, CH), 51.9 (*J* = 5.7, *C*H_3_O), 71.8 (*J* = 6.3, CH_2_O); ^1^H-NMR (CDCl_3_) δ 0.89 (d, *J_HH_* = 6.8, 6H, C*H*_3_CH), 1.84–1.96 (m, 1H, CH), 3.71 (d, *J_PH_* = 11.9, 3H, CH_3_O) 3.74–3.83 (m, 2H, CH_2_O), 6.72 (d, *J_PH_* = 695.8, 1H, PH).

*Methyl pentyl H-phosphonate* (**2d**): Yield: 50%; ^31^P-NMR and HRMS (see Table 7); ^13^C-NMR (CDCl_3_) δ 13.9 (*C*H_3_CH_2_), 22.2 (CH_3_*C*H_2_), 27.6 (CH_3_CH_2_*C*H_2_), 30.1 (*J* = 6.2, *C*H_2_CH_2_O), 51.9 (*J* = 5.7, *C*H_3_O), 67.0 (*J* = 6.1, CH_2_O); ^1^H-NMR (CDCl_3_) δ 0.91 (t, *J* = 6.9, 3H, C*H*_3_CH_2_), 1.29–1.44 (m, 4H, C*H*_2_C*H*_2_CH_3_), 1.63–1.78 (m, 2H, C*H*_2_CH_2_O), 3.78, (d, *J_PH_* = 11.9, 3H, CH_3_O) 3.99–4.17 (m, 2H, CH_2_O), 6.79 (d, *J_PH_* = 695.2, 1H, PH).

*Ethyl propyl H-phosphonate* (**5b**): Yield: 30%; ^31^P-NMR and HRMS (see Table 7); ^13^C-NMR (CDCl_3_) δ 10.0 (*C*H_3_CH_2_CH_2_), 16.3 (*J* = 6.1, *C*H_3_CH_2_O), 23.8 (*J* = 6.4, CH_3_*C*H_2_CH_2_), 61.8 (*J* = 5.8, CH_2_*C*H_2_O), 67.3 (*J* = 6.0, CH_3_*C*H_2_O); ^1^H NMR (CDCl_3_) δ 0.96 (t, *J_HH_* = 7.4, 3H, C*H*_3_CH_2_CH_2_), 1.35 (t, *J_HH_* = 7.1, 3H, C*H*_3_CH_2_O), 1.63–1.78 (m, 2H, CH_3_C*H*_2_CH_2_), 3.94–4.07, (m, 2H, CH_2_C*H*_2_O) 4.07–4.21 (m, 2H, CH_3_C*H*_2_O), 6.80 (d, *J_PH_* = 692.4, 1H, PH).

*i-Butyl ethyl H-phosphonate* (**5c**): Yield: 36%; ^31^P-NMR and HRMS (see Table 7); ^13^C-NMR (CDCl_3_) δ 16.3 (*J* = 6.2, *C*H_3_CH_2_), 18.7 (*J* = 1.7, *C*H_3_CH), 29.1 (*J* = 6.5, CH), 61.8 (*J* = 5.7, CH_3_*C*H_2_O), 71.6 (*J* = 6.2, CH*C*H_2_O); ^1^H NMR (CDCl_3_) δ 0.96 (d, *J_HH_* = 6.7, 6H, C*H*_3_CH), 1.37 (t, *J_HH_* = 7.1, 3H, C*H*_3_CH_2_), 1.89–2.06 (m, 1H, CH), 3.77–3.91, (m, 2H, CHC*H*_2_O) 4.09–4.22 (m, 2H, CH_3_C*H*_2_O), 6.82 (d, *J_PH_* = 692.7, 1H, PH).

## 4. Conclusions

In summary, a MW-assisted continuous flow method was developed for the catalyst-free alcoholysis of dialkyl *H*-phosphonates. By the optimization of the reaction parameters, the alcoholysis was fine-tuned towards dialkyl *H*-phosphonates with two different or with two identical alkyl groups. The selectivity of the reaction was similar in the flow and batch approaches; however, the continuous protocol offered several advantages. The dialkyl *H*-phosphonates with two different alkyl groups can be obtained at a shorter reaction time and in a higher ratio. Furthermore, a somewhat higher productivity (0.8–1.0 g/h) can be attained by the continuous flow method. Five new dialkyl *H*-phosphonates with different alkyl groups were isolated and characterized, which may be valuable building blocks in the synthesis of chiral organophosphorus compounds.

## Figures and Tables

**Table 1:** Synthetic methods for the preparation of dialkyl *H*-phosphonates bearing two different alkyl groups.

Entry	Reaction	Average Yield (%)	Flow Compatible	Ref.
**1**	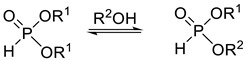	30–50	+	[46,50,52]
**2**	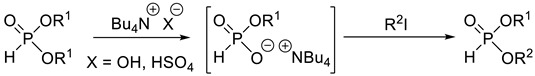	50–70	−	[62,63]
**3**	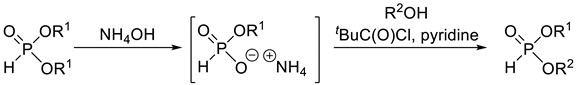	50–85	−	[64]
**4**	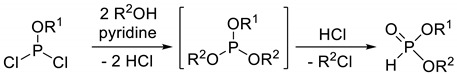	30–60	−	[65]

**Table 2:**
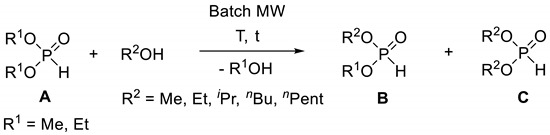
Alcoholysis of dialkyl *H*-phosphonates in a batch microwave (MW) reactor [33].

Entry	R^1^	R^2^	R^2^OH (equiv)	T (°C)	t (min)	Composition (%) ^a^		
						A	B	C
**1**	Me	Et	25	100	120	22	**56**	22
**2**	Me	Et	50	175	40	0	4	**96**
**3**	Me	*^n^*Bu	25	125	60	0	**40**	60
**4**	Me	*^n^*Bu	50	150	60	0	0	**96**
**5**	Et	Me	25	125	120	49	**38**	13
**6**	Et	Me	50	175	40	0	21	**79**
**7**	Et	*^i^*Pr	25	125	60	26	**57**	17
**8**	Et	*^i^*Pr	50	175	40	0	6	**94**
**9**	Et	*^n^*Bu	25	125	60	25	**54**	21
**10**	Et	*^n^*Bu	50	175	40	0	2	**98**
**11**	Et	*^n^*Pent	25	125	60	3	**52**	45
**12**	Et	*^n^*Pent	50	175	40	0	8	**92**

^a^ Analyzed by GC. Bold numbers indicate the target compounds in the reactions.

**Table 3:**
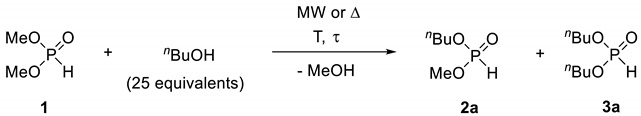
Continuous flow alcoholysis of DMP with *^n^*BuOH.

Entry	Mode of Heating	Power (W)	T (°C) ^a^	Flow Rate	τ (min)	Conversion (%)	Composition (%) ^c^			Yield (%) ^d^
				(mL/min) ^b^			1	2a	3a	
**1**	MW	22	100	1.4	5	26	74	25	1	-
**2**	MW	14	100	0.45	15	52	48	44	8	-
**3**	MW	10	100	0.25	30	65	35	**53**	12	48 (**2a**)
**4**	MW	8	100	0.15	45	76	24	50	26	-
**5**	MW	5	100	0.10	60	83	18	47	35	-
**6**	Δ	-	100	0.25	30	15	85	15	0	-
**7**	MW	18	125	0.25	30	93	7	43	50	-
**8**	MW	38	150	0.25	30	100	0	22	78	-
**9**	MW	59	175	0.25	30	100	0	9	**91** ^e,f^	88 (**3a**)
**10**	Δ	-	175	0.25	30	98	2	15	83	-

^a^ The pressure was 17 bar; ^b^ Based on the HPLC pump; ^c^ the mixtures from the stationary operation were analyzed by GC; ^d^ after column chromatography; ^e^ No change on longer residence time (45 min); ^f^ No change on higher excess (50 equiv) of *^n^*BuOH. Bold numbers indicate the target compounds in the reactions.

**Table 4:**
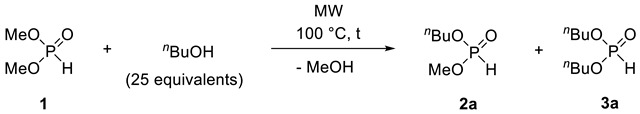
Comparative experiments for the alcoholysis of dimethyl *H*-phosphonate (DMP) with *^n^*BuOH in a batch MW reactor.

Entry	t (min)	Conversion (%)	Composition (%) ^a^		
			1	2a	3a
**1**	5	24	76	20	4
**2**	15	46	54	37	9
**3**	30	59	41	47	12
**4**	45	67	33	49	18
**5**	60	73	26	45	29

^a^ Based on GC.

**Table 5:**
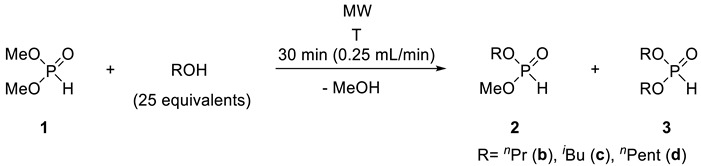
Continuous flow alcoholysis of DMP with various alcohols.

Entry	R	Power (W)	T (°C) ^a^	Composition (%) ^b^			Yield (%) ^c^
				1	2	3	
**1**	*^n^*Pr	10	100	45	**46**	9	42 (**2b**)
**2**		57	175	0	10	**90**	85 (**3b**)
**3**	*^i^*Bu	10	100	38	**51**	11	46 (**2c**)
**4**		71	175	0	10	**90**	86 (**3c**)
**5**	*^n^*Pent	10	100	32	**55**	13	51 (**2d**)
**6**		55	175	0	8	**92**	88 (**3d**)

^a^ The pressure was 17 bar; ^b^ the mixtures from the stationary operation were analyzed by GC; ^c^ after column chromatography. Bold numbers indicate the target compounds in the reactions.

**Table 6:**
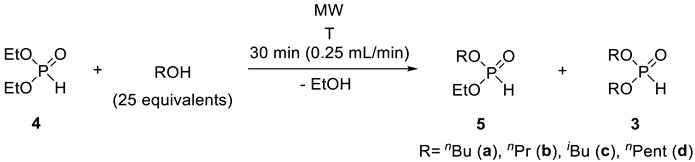
Continuous flow alcoholysis of DEP with alcohols.

Entry	R	Power (W)	T (°C) ^a^	Composition (%) ^b^			Yield (%) ^c^
				4	5	3	
**1**	*^n^*Bu	10	100	58	34	8	-
**2**		17	125	44	**42**	14	38 (**5a**)
**3**		38	150	7	32	61	-
**4**		57	175	0	11	**89 ^d,e^**	85 (**3a**)
**5**	*^n^*Pr	18	125	51	**41**	8	36 (**5b**)
**6**		54	175	4	22	74	–
**7**		83	200	2	14	**84 ^d,e^**	78 (**3b**)
**8**	*^i^*Bu	21	125	21	**40**	39	36 (**5c**)
**9**		63	175	3	26	71	–
**10**		105	200	0	16	**84 ^d,e^**	77 (**3c**)
**11**	*^n^*Pent	10	125	31	**44**	25	40 (**5d**)
**12**		39	175	2	13	**85 ^d,e^**	80 (**3d**)

^a^ The pressure was 17 bar; ^b^ the mixtures from the stationary operation were analyzed by GC; ^c^ after column chromatography; ^d^ no change upon longer residence time (45 min); ^e^ no change upon higher excess (50 equiv) of *^n^*BuOH. Bold numbers indicate the target compounds in the reactions.

**Table 7:** ^31^P-NMR and mass data for the dialkyl *H*-phosphonates prepared.

Compound	δ_P_ (CDCl_3_)	δ_P_ [Ref]	[M + H]^+^_found_	[M + H]^+^_requires_
**2a**	9.3 (695.0)	9.3 [52]	153.0684	153.0681
**2b**	9.3 (695.4)	‒	139.0522	139.0523
**2c**	6.7 (695.7)	‒	153.0676	153.0681
**2d**	6.8 (695.2)	‒	167.0833	167.0837
**3a**	7.8	7.9 [66]	195.1153	195.1150
**3b**	7.9	7.8 [67]	167.0833	167.0837
**3c**	8.1	8.0 [67]	195.1145	195.1150
**3d**	7.8	8.1 [68]	223.1462	223.1463
**5a**	7.6 (693.0)	7.6 [52]	167.0840	167.0837
**5b**	8.6 (692.4)	‒	153.0673	153.0681
**5c**	7.8 (692.8)	‒	167.0832	167.0837
**5d**	7.7 (692.1)	7.7 [52]	181.0992	181.0994
